# Variations in emergency care for severe pre-eclampsia in Uganda: a national evaluation study

**DOI:** 10.1016/j.xagr.2024.100424

**Published:** 2025-01-13

**Authors:** Annettee Nakimuli, Jackline Akello, Musa Sekikubo, Sarah Nakubulwa, Moses Adroma, Rehema Nabuufu, Emmanuel Obuya, John Paul Bagala, Andrew Twinamatsiko, Hadijah Nakatudde, Patrica Pirio, Grace Latigi, Baifa Arwinyo, Kenneth Mugabe, Irene Chebet, Richard Mugahi, Isabella Aitchison, Charlotte Patient, Ashley Moffett, Catherine E Aiken

**Affiliations:** 1 Department of Obstetrics and Gynaecology, School of Medicine, Makerere University, Kampala, Uganda (Nakimuli, Akello, Sekikubo, Nakubulwa, Adroma); 2 Department of Obstetrics and Gynaecology, Kawempe National Referral Hospital, Kampala, Uganda (Nakimuli, Akello, Sekikubo, Nakubulwa, Adroma, Nabuufu); 3 School of Public Health, Makerere University, Kampala, Uganda (Obuya); 4 Ministry of Health Headquarters, Kampala, Uganda (Bagala, Twinamatsiko, Nakatudde, Mugahi); 5 UNICEF Uganda Office (Pirio, Latigi), Kampala, Uganda; 6 Department of Obstetrics and Gynaecology, Gulu Regional Referral Hospital, Gulu, Uganda (Arwinyo); 7 Department of Obstetrics and Gynaecology, Mbale Regional Referral Hospital, Mbale, Uganda (Mugabe); 8 Department of Obstetrics and Gynaecology, Soroti Regional Referral Hospital, Soroti, Uganda (Chebet); 9 Department of Obstetrics and Gynaecology, University of Cambridge, The Rosie Hospital and NIHR Cambridge Biomedical Research Centre, Cambridge, United Kingdom (Aitchison, Patient, Aiken); 10 Department of Pathology and Centre for Trophoblast Research, University of Cambridge, Cambridge, United Kingdom (Moffett)

**Keywords:** Maternal health, multidisciplinary, obstetrics, pre-eclampsia, quality improvement, resources, Uganda

## Abstract

**Background:**

Worldwide, 70% of maternal deaths occur in Sub-Saharan Africa. Approximately 10% are attributable to hypertensive disorders of pregnancy, primarily complications of pre-eclampsia. Timely and effective care improves outcomes, but this is not consistently available, particularly in low-resource settings such as Uganda.

**Objectives:**

We conducted a national evaluation of the provision of prompt and safe care for women with severe pre-eclampsia across all regions of Uganda. We explored the wider health system-related factors, eg supply availability, facilities, and emergency training drills, that may affect the ability of healthcare facilities to deliver optimal pre-eclampsia care.

**Study design:**

A multidisciplinary research team carried out in-person, unannounced visits to maternity facilities across Uganda to assess the quality of care provided. Evaluations of facilities, staff interviews, and case notes reviews were performed.

**Results:**

75 maternity facilities were included from all regions of Uganda. Of these, 25% were unable to provide correct emergency care for severe pre-eclampsia, and 21% were unable to consistently provide delivery or referral for eclamptic seizure within 12 hours. Factors strongly associated with not providing optimal pre-eclampsia care were lack of staff training, lack of readily available clinical protocols, lack of antenatal education, lack of close postnatal monitoring and care that was not always woman-centered.

**Conclusions:**

The key barriers associated with delayed or poor quality pre-eclampsia care across Uganda are potentially modifiable with strengthened clinical governance initiatives. Developing context-specific, standardized, national training and educational programmes could be effective in reducing rates of maternal and neonatal morbidity and mortality from pre-eclampsia.

**AJOG Global Reports at a Glance**

**Why was this study conducted?**

Pre-eclampsia is a life-threatening obstetric condition where prompt and appropriate care can save maternal and neonatal lives. Understanding the factors that influence the provision of pre-eclampsia care across Uganda may help identify opportunities for improvement.

**Key findings**

25% of maternity facilities were unable to provide correct emergency care for severe pre-eclampsia. 21% of facilities were unable to consistently provide delivery or referral in cases of eclamptic seizure within 12 hours. Failure to provide correct and timely emergency care was strongly associated with lack of multidisciplinary team training for treating pre-eclampsia.

**What does this add to what is known?**

We identify the key barriers to providing safe and timely pre-eclampsia care across Uganda. Regular staff training and readily available protocols are associated with a greater likelihood of pre-eclampsia care being delivered in a safe and timely manner. Interventions to improve clinical leadership and governance may be effective in reducing rates of maternal and neonatal morbidity and mortality.

## Introduction

Approximately 10% of maternal deaths worldwide are attributable to hypertensive disorders of pregnancy, which are primarily concentrated in Sub-Saharan Africa.^1^ Pre-eclampsia is common in African women, affecting around 10% of all pregnancies.^2^ Previous work in Uganda suggests that pre-eclampsia is a factor in up 25% of all maternal deaths^3^ and is also a major cause of morbidity and mortality in neonates.^4^

Severe pre-eclampsia is managed via a series of well-established interventions designed to promote survival of the mother and baby, and to prevent serious morbidity. The initial priority should be stabilization of the mother, which includes administration of intravenous magnesium sulphate to lower seizure threshold and antihypertensive medication to reduce the risk of stroke. In most cases, stabilization of the mother should be followed rapidly by delivery of the baby. When appropriate care is provided promptly and effectively the outcomes of severe pre-eclampsia for both mother and baby are significantly improved.^5^ However, providing all the elements of appropriate pre-eclampsia care on an emergency basis can present a significant challenge, especially in high-acuity low-resource maternity settings. Our previous work in urban Uganda illustrates the challenges of timely delivery^6^ and the barriers that clinicians perceive to providing prompt and safe care for women with pre-eclampsia^7^ in this setting.

Many interventions have been trialed globally to improve pre-eclampsia care on a healthcare system level.^8^, ^9^, ^10^ However, our previous work suggests the need for a systematic evaluation of the gaps in care specifically within the Ugandan context^7^ to determine which strategies would be most effective at preventing maternal deaths from pre-eclampsia across Uganda.

We aimed to explore how pre-eclampsia care is currently provided across a sample of sites drawn from all regions of Uganda, taking a national approach to defining the key challenges inherent in providing prompt and safe emergency care for severe pre-eclampsia.

## Materials and methods

Data were collected as part of the Reduction in Maternal and Neonatal Mortality Related to Pre-eclampsia & Eclampsia (REAP) study. We conducted assessments in medical facilities across Uganda during 2021. Care was benchmarked against standards drawn from the WHO^11^ and Ugandan National Guidelines developed by the Ministry of Health.^12^

### Study sites

All 21 national and regional maternity referral hospitals (designated here ‘specialist hospitals’) in Uganda were included in the study. Within the catchment of each of these 21 specialist hospitals, a non-probabilistic convenience sampling procedure was used to choose a general hospital, a clinic providing specialist obstetric care, and a clinic not providing specialist obstetric care. A private not-for-profit facility within the catchment of each specialist hospital was also selected for study. Where possible all of these facility types were included, although in practice not all types of facility existed within each of the 21 catchment areas, giving a total sample size of 75 institutions. The latitude, longitude, and altitude of each facility was calculated from publicly available data-sources.

### Assessment teams

A research group of 48 experienced Ugandan maternity care professionals was recruited from across the country. Each individual research team consisted of 3 people: an obstetrician, medical officer, and a midwife. All team members participated in a full day of training prior to the commencement of data collection, including specific training on the data collection software and tools. As data collection was disrupted by restrictions related to the COVID-19 pandemic, a further 2-hour online refresher training was given after restrictions were lifted.

### Study conduct

Each multidisciplinary research team was assigned to visit selected healthcare facilities (no researcher was assigned to evaluate their own facility). The health facilities were not informed in advance about the visit. Each research team carried an introductory letter from the Ministry of Health (MOH), which was addressed to the manager of each health facility and included details of the planned assessments. Upon arrival at the facility, the assessors discussed the study with the managers of the facility and then proceeded with the assessment. In all visits, the assessment was conducted as planned.

### Assessment procedures

Several forms of assessment were performed during the visit to each facility. These included assessment of (1) written policies, guidelines, and protocols, (2) clinical logs if available, recording the number of complications and outcomes, (3) a sample of up to 10 patient files from recent cases of severe pre-eclampsia, and (4) observation of clinical areas (delivery unit, antenatal and postnatal areas), practices, and equipment. The assessments of policies and patient files were performed independently by different team members during the same site visit. The assessment team aimed to be minimally disruptive to the operation of the facility. Where a facility did not have 10 relevant recent severe pre-eclampsia cases with medical records available for assessment, a smaller number of cases was assessed. Pre-eclampsia was diagnosed according to local modifications of internationally accepted criteria.^13^ The definition of pre-eclampsia varied slightly by site according to availability of diagnostic resources, for example urine dipsticks and laboratory facilities. The research team used prespecified criteria to evaluate whether the rights of women (access to care, information, emotional support, women-centered care, dignity and respect, privacy, companionship) were respected, which included assessing facilities, privacy options during examinations, and discussions with women undergoing care and staff.

### Assessment of correct emergency management

At each facility, up to 10 patient files were assessed by the team to determine whether patients diagnosed with severe pre-eclampsia received gold standard care: we defined this as prompt loading doses of 14g magnesium sulphate (4g intravenous and 10g intramuscular) and appropriate antihypertensive treatment, followed by arrangements for either prompt transfer or delivery, depending on the capability of the facility, made as soon as the mother was sufficiently stable.^12^ The timeframe to effect either delivery or transfer to a suitable facility for delivery was pragmatically determined based on our previous work as 12 hours from eclamptic seizure for this very high-risk cohort of women.^6^^,^^7^ Appropriate hypertensive therapy in this context was defined as any drug considered by the WHO to be appropriate for use in pregnancy to lower blood pressure, for example labetalol, methyldopa, nifedipine, hydralazine.

### Data analysis

Data were collected at each site using a locally-modified version of the WHO Pre-eclampsia Quality Improvement Tool data collection (Supplementary Appendix 1) via Open Data Kit (ODK)^14^ on electronic tablets. Descriptive statistics were used to summarize our findings (number and % for categorical outcomes, median and interquartile range for numerical outcomes). Chi-square testing was conducted to check whether there were differences in unadjusted analysis. Multivariate logistic regression models were constructed to adjust for relevant variables, including the level of care each facility provided, geographical region, and funding model (public v private). Analyses was performed using R for statistical computing v4.2.2.^15^

### Ethics statement

The study was approved by the School of Medicine Research and Ethics Committee (Ref number Mak-SOMREC-2021-140). The approval included a waiver of consent to align to the study procedures.

## Results

**Figure 1:**
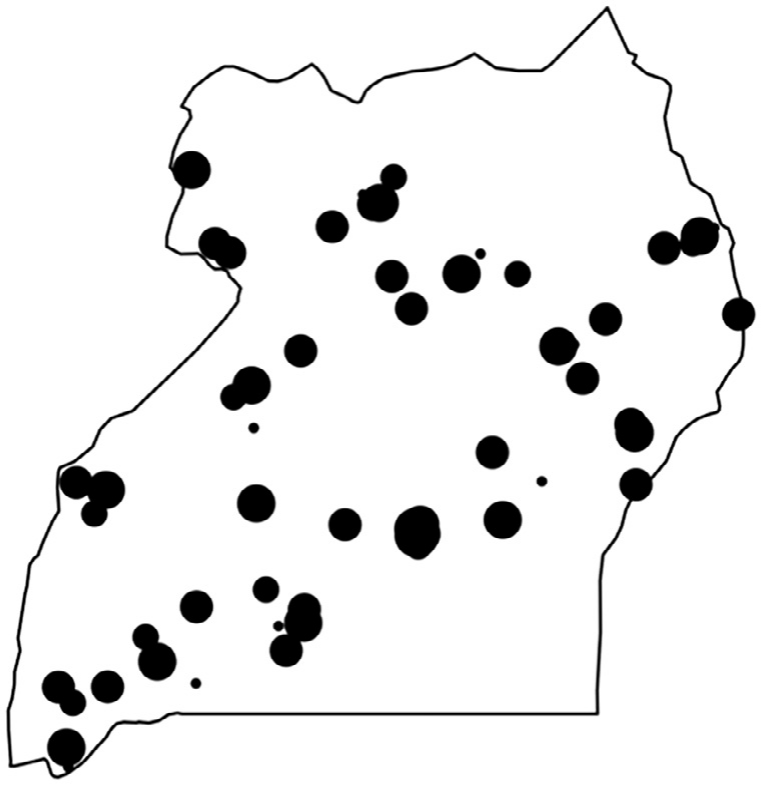
Map of Uganda showing locations of all facilities providing maternity care included in the study The size of the facility is represented by the size of the circle (largest circles; specialist hospitals, smallest circles; clinics with general expertise).

The 75 healthcare facilities included in the study covered all 4 regions of Uganda. Of 135 administrative districts within Uganda, 43 (32%) were represented. The geographical spread included both coastal and mountainous regions, as well as peri-urban areas (Figure 1). We included 45 (60%) publicly-funded and 30 (40%) privately-funded facilities. All levels of facility within the Ugandan health care system were represented: specialist hospitals; n=21 (28%), general hospitals; n=25 (33%), health centers with obstetric care; n=15 (20%) and health center with general care; n=14 (19%).

Nakimuli. Variations in emergency care for severe pre-eclampsia in Uganda. AJOG Glob Rep 2024.

**Figure 2:**
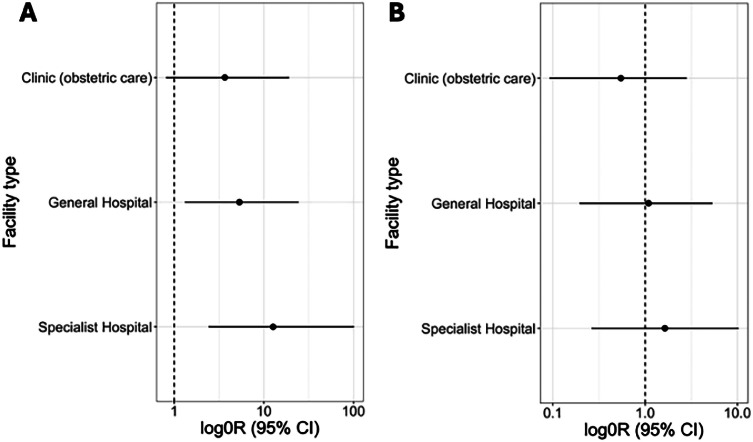
A, Odds ratio for providing correct emergency care for severe pre-eclampsia, by level of facility B, Odds ratio for providing emergency transfer or delivery within 12 hours for eclampsia, by level of facility. Points represent the odds ratios derived from logistic regression models adjusted for region of Uganda and funding model (public v. private). Lines represent 95% confidence intervals around the point estimate of the odds ratio. The referent category is health care clinics that provide general medical care only. Odds ratios and 95% confidence intervals are displayed on a log-transformed x-axis.

Correct emergency management for severe pre-eclampsia was initiated in 75% of facilities (56/75). In facilities where correct management was not initiated, 6/19 facilities did not give magnesium sulphate according to international guidelines^11^ and 4/19 did not give magnesium sulphate prior to transfer. Correct management was significantly more likely at more specialized health care facilities (Figure 2A). Delivery or transfer within 12 hours of eclamptic seizure was achieved in 79% (59/75) cases. There was no difference in the chance of achieving prompt delivery or transfer relating to level of care (Figure 2B). There were no variations by funding model, region or by altitude of the facilities in their ability to delivery correct or prompt emergency management for severe pre-eclampsia.

Nakimuli. Variations in emergency care for severe pre-eclampsia in Uganda. AJOG Glob Rep 2024.

Clinical aspects of care that were associated with a greater likelihood of providing correct emergency management for severe pre-eclampsia were the immediate availability of clinical protocols in the labor and delivery area (p=0.02, Table 1) and evidence of regular multidisciplinary team pre-eclampsia drills (p=0.002, Table 1). Availability of supplies of drugs and skilled staff emerged as potential factors increasing the likelihood of high-quality care in unadjusted analysis. However after adjustment for the level of the healthcare facility, there was no association between providing correct emergency management and logistical factors such as drugs being frequently out of stock, having skilled staff, or emergency trolleys not being well-maintained (Table 1).

**Table 1:** Clinical and logistical aspects of care

	Unadjusted OR (95%CI)	*P* value	Adjusted OR (95% CI)	*P* value
Availability of pre-eclampsia guidelines in labor area	**4.7 (1.6–4.8)**	**.006**	**3.9 (1.2–13.7)**	**.02**
Supplies of emergency drugs for pre-eclampsia	3.0 (1.1–9.1)	.04	3.3 (0.9–1.2)	.06
Well-stocked emergency trolley for hypertensive crisis	2.8 (0.9–8.3)	.06	2.2 (0.7–7.3)	.2
Staff with skills to manage emergency	4.7 (1.5–15.3)	.008	3.1 (0.9–11.4)	.08
Regular multidisciplinary team emergency drills	**10.4 (3.0–48.8)**	**< .001**	**10.1 (2.6–52.9)**	**.002**

Likelihood of meeting standards related to each factor in facilities that provide correct emergency care for severe pre-eclampsia compared to those that did not provide correct emergency care for severe pre-eclampsia (referent group).

Unadjusted odds ratios and 95% confidence intervals are presented alongside adjusted odds ratios and 95% confidence intervals, where the co-variates included in the model are level of care provided by the facility and funding model (public v private). Results with *P*<.05 in adjusted analysis are highlighted in bold.

Factors related to nursing and anesthetic care were also assessed for association with correct emergency management of pre-eclampsia (Table 2). In adjusted analysis, the provision of close monitoring for women with severe pre-eclampsia postnatally was associated with a greater likelihood that women received correct emergency management for pre-eclampsia (*P*=.04; Table 2), however this association was not observed for antenatal monitoring in adjusted analysis.

**Table 2:** Nursing and anesthetic aspects of care

	Unadjusted OR (95%CI)	*P* value	Adjusted OR (95% CI)	*P* value
Frequent monitoring of vital signs	2.7 (0.8–12.8)	.14	2.5 (0.6–13.8)	.3
Fluid balance tracking	4.9 (0.8–9.3)	.13	3.0 (0.5–8.8)	.3
Monitoring immediately after delivery	**5.9 (1.9–20.7)**	**.03**	**4.9 (1.1–24.0)**	**.04**
Provision of spinal anesthetic	4.4 (1.3–14.6)	.01	NA	NA
Provision of general anesthetic	3.0 (0.9–9.3)	.06	NA	NA

Likelihood of meeting standards related to each factor in facilities that provide correct emergency care for severe pre-eclampsia compared to those that did not provide correct emergency care for severe pre-eclampsia (referent group).

Unadjusted odds ratios and 95% confidence intervals are presented alongside adjusted odds ratios and 95% confidence intervals, where the co-variates included in the model are level of care provided by the facility and funding model (public v private). Results with p<0.05 in adjusted analysis are highlighted in bold.

The adjusted OR for anesthesia provision could not be calculated as these were not available in any nonspecialist clinic-based facility.

Correct emergency management for pre-eclampsia was more commonly performed in facilities where the rights of women were consistently respected, including ensuring dignity, privacy and emotional support during labor and delivery (*P*=.02; Table 3). There was also a strong association between women having access to antenatal education about pre-eclampsia and a greater likelihood of receiving correct emergency care (*P*=.004; Table 3). There was no association between likelihood of receiving correct emergency care and the frequency of antenatal visits more generally or education about long-term implications of pre-eclampsia (Table 3).

**Table 3:** Holistic and women-centered aspects of care

	Unadjusted OR (95%CI)	*P* value	Adjusted OR (95% CI)	*P* value
Respect and dignity for patients	**3.8 (1.0–14.3)**	**.04**	**6.9 (1.5–38.9)**	**.02**
Antenatal education on pre-eclampsia	**3.9 (1.3–12.3)**	**.01**	**7.1 (2.0–30.1)**	**.004**
Frequency of antenatal clinic visit	0.6 (0.2–2.2)	.43	1.3 (0.3–6.4)	.76
Postnatal follow-up and contact	7.4 (1.9–49.2)	.01	5.2 (1.2–3.7)	.05
Education on longer-term health	4.0 (1.0–27.1)	.08	3.1 (0.7–22.8)	.20

Likelihood of meeting standards related to each factor in facilities that provide correct emergency care for severe pre-eclampsia compared to those that did not provide correct emergency care for severe pre-eclampsia (referent group).

Unadjusted odds ratios and 95% confidence intervals are presented alongside adjusted odds ratios and 95% confidence intervals, where the co-variates included in the model are level of care provided by the facility and funding model (public v private). Results with p<0.05 in adjusted analysis are highlighted in bold.

### Principle findings

Safe and prompt emergency management for severe pre-eclampsia was provided in the majority of facilities that we studied across Uganda. However a substantial minority (25%) did not provide all basic aspects of care. Delivery or transfer following eclamptic seizure could not be achieved promptly in 21% of facilities. The factors most strongly associated with greater likelihood of successfully delivering emergency pre-eclampsia care at facility level were mainly aspects of (1) good clinical governance and (2) providing respectful and informative care to women.

### Clinical implications

Maternal mortality in Sub-Saharan Africa is often understood in the context of the ‘3 Delays’ model.^16^ We focused on factors that influence the third delay, at facility level, specifically factors that increase the likelihood of receiving prompt and safe emergency care with respect to severe pre-eclampsia. We found that the likelihood of receiving prompt and effective care was strongly associated with organizational and cultural factors within the facility. Women who attended facilities with more capability for obstetric care (specialist hospitals) were more likely to receive all essential elements of emergency pre-eclampsia care (magnesium sulphate, antihypertensives and delivery planning), but were not more likely to achieve delivery within a 12 hours target following eclamptic seizure. This is in keeping with our previous work, which demonstrated that although clinical decision-making is excellent, the workload in specialist maternity facilities in Uganda is often overwhelming and can impede timely care.^6^

Two major factors related to clinical governance were identified as strongly associated with an increased likelihood that women received safe and prompt emergency care, regardless of the facility they attended. These were (1) the immediate availability of clinical protocols for treating pre-eclampsia within the relevant clinical area and (2) multidisciplinary team training in emergency pre-eclampsia care. The effectiveness of training in obstetric emergency care has been studied across global contexts and emerges as a key intervention that reduces maternal and neonatal mortality.^17^ In various global settings, initiatives have been developed to standardise this type of training on a national basis.^18^^,^^19^ Our results suggest that the development of a Uganda-specific standard multidisciplinary team training module for obstetric emergencies, suitable for implementation at facilities of any level, could be effective at improving the delivery of prompt and safe pre-eclampsia care. Training and protocol availability may be interlinked, as evidence suggests that effective training increases the likelihood that protocols will be followed effectively when emergencies actually arise.^20^ Our previous qualitative studies of healthcare professionals providing pre-eclampsia care in Kampala suggest that clinical decision-making would be supported by implementing these measures.^7^ Both ensuring consistent protocol availability and implementing regular training require clear and effective clinical leadership and understanding of clinical governance.

We found that provision of prompt and effective emergency care prior to delivery was strongly associated with close monitoring of affected women following their delivery. This association is clearly not causal, but instead likely reflects facilities that provide an overall high standard of care. In Uganda, 36% of maternal deaths occur in the immediate postnatal period, approximately 28% of which are attributable to pre-eclampsia.^3^ Hence monitoring throughout the perinatal period is crucial, yet often overlooked. The strong association between settings where robust postnatal monitoring occurs and where effective antenatal emergency care is provided likely indicates the leadership of clinicians who understand the risks and natural history of severe pre-eclampsia.

The final element that was strongly associated with good emergency management was the provision of respectful and informative maternity care. This is a crucial element of maternity care in all settings, however particularly in high-acuity settings where the priority is to save maternal and neonatal life, aspects of woman-centered care can be overlooked. We found that facilities that provided good quality emergency care were also more likely to provide dignified and respectful care. We assessed a number of elements encompassed by this concept, including whether women had good access to emotional support, and whether the care provided respected her dignity, privacy, and need for companionship during acute admissions. The association between woman-centered care and care that meets appropriate clinical standards is a critical finding that adds to the weight of argument that dignity and respect should be central to all maternity care provided both across Uganda and globally.^21^

### Research implications

Future work should involve research into how context-appropriate clinical leadership and training courses might be co-produced between Ugandan maternity care professionals and researchers. In other contexts, nationally standardized hands-on multidisciplinary training for obstetric emergencies has proven successful.^17^ It is a key research goal to investigate how this kind of initiative could be developed in the Ugandan context, possibly within a centralized maternity research institute.

### Strengths and limitations

A key strength of our study is adopting a nation-wide approach to identifying opportunities for improving emergency care for pre-eclampsia. However, despite including a sample of maternity units from across Uganda (Figure 1), there are likely to be local and regional factors that influence care provision that are not identifiable in our study. Cultural and geographical issues that are unique to specific areas will require individualized solutions to address the challenges they pose. Instead, we have sought to make an objective contribution to understanding pre-eclampsia management in the broader Ugandan context and seeking potential opportunities for improvement on a national basis. One potential limitation of our study design is that differing factors might have emerged had facilities been evaluated via other methodologies, for example a survey-based study or in-depth interviews with professionals in each setting. However, the face-to-face evaluations that we conducted allowed the research teams to develop personal and nuanced understanding of the context of each facility, which was a major strength of the study. The study design of unannounced visits was important to ensure that we captured the day-to-day operation of the facilities rather than a ‘special’ mode of working during the visits, however a limitation of this design may have been that staff were less forthcoming with unexpected visitors than they might have been in the context of a prearranged research visit.

## Conclusions

Hypertensive disorders of pregnancy contribute to approximately 1-in-4 maternal deaths in urban Uganda.^3^ Following our previous work, we identified barriers to providing high quality pre-eclampsia emergency care that relate closely to how maternity services are governed and the culture that exists within them.^7^ While factors such as obstetric drug availability impacted care at smaller, less-specialized facilities, leadership-related barriers to providing excellent emergency pre-eclampsia care were present at all types of facilities, including those that were privately funded. We identify organizational and training needs for healthcare professionals to improve and standardize the delivery of emergency care for pre-eclampsia across Uganda. We suggest that national-level initiatives for maternity leadership and training will be central to addressing these needs.

## CRediT authorship contribution statement

**Annettee Nakimuli:** Writing – original draft, Visualization, Validation, Supervision, Methodology, Investigation, Funding acquisition, Formal analysis, Conceptualization. **Jackline Akello:** Writing – review & editing, Investigation. **Musa Sekikubo:** Writing – original draft, Formal analysis, Conceptualization. **Sarah Nakubulwa:** Writing – review & editing, Investigation. **Moses Adroma:** Writing – review & editing, Investigation. **Rehema Nabuufu:** Writing – review & editing, Investigation. **Emmanuel Obuya:** Writing – review & editing, Formal analysis, Data curation. **John Paul Bagala:** Writing – review & editing, Investigation. **Andrew Twinamatsiko:** Writing – review & editing, Investigation. **Hadijah Nakatudde:** Writing – review & editing, Investigation. **Patrica Pirio:** Writing – review & editing, Investigation. **Grace Latigi:** Writing – review & editing, Investigation. **Baifa Arwinyo:** Writing – review & editing, Investigation. **Kenneth Mugabe:** Writing – review & editing, Investigation. **Irene Chebet:** Writing – review & editing, Investigation. **Richard Mugahi:** Writing – review & editing, Investigation. **Isabella Aitchison:** Writing – original draft, Formal analysis. **Charlotte Patient:** Writing – review & editing, Formal analysis. **Ashley Moffett:** Writing – original draft, Formal analysis. **Catherine E Aiken:** Writing – original draft, Visualization, Validation, Supervision, Formal analysis, Data curation, Conceptualization.

## Appendix Supplementary materials

Image, application 1

## Footnotes

Supplementary material associated with this article can be found in the online version at doi:10.1016/j.xagr.2024.100424.
