# Supplementary material for: Variations in emergency care for severe pre-eclampsia in Uganda: a national evaluation study
Source: AJOG Glob Rep. 2025 Jan 13;5(1):100424. doi: 10.1016/j.xagr.2024.100424 (PMC11833625; doi:10.1016/j.xagr.2024.100424)
Supplement: Supplementary file 1 [file mmc1.docx]

PRE-ECLAMPSIA AND ECLAMPSIA

**Objective:** To assess whether women with pre-eclampsia or eclampsia promptly receive appropriate interventions with quality standards.

**Sources of data and instructions:**

- Written policies, guidelines, and protocols
- Clinical logs: number of complications and outcomes – notice if there are patterns/stereotypes which can indicate low quality of care
- Patient files: review 10 files of pre-eclampsia and eclampsia randomly chosen from the last three months. *Review all files of maternal death from the last 12 months*
- Observation of practices, equipment and its use in admission area, labour and birth area, postnatal area. Give observation during medical round in the ward a priority, ask permission of the women to check files, ensure “non-intrusive” observations, with a focus on pre-eclampsia and eclampsia patients.
- Structured interviews with women and staff
- *Please circle “Y” for “YES” and “N” for “NO” and note your observations in the comment column*

| **The hospital has adequate resources and organization to manage preeclampsia and eclampsia** | **Response** | **Comments** |
| --- | --- | --- |
| 1. The health facility has written, up-to-date clinical protocols on the management of pre-eclampsia and eclampsia that are available in the ANC, labor, childbirth and postnatal areas of the maternity unit and are consistent with WHO guidelines | Y N |  |
| 1. The health facility has sufficient supplies of oral and intravenous antihypertensive agents and magnesium sulfate available in sufficient quantities at all times in the antenatal, labour and childbirth areas of the maternity unit | Y N |  |
| 1. A kit is ready for eclampsia and staff skilled enough to manage the emergency are readily available 24h /7 days | Y N |  |
| 1. Health-care staff in the maternity unit receive in-service – bedside training and regular refresher sessions in the use of antihypertensive agents, IV infusion and magnesium sulfate for treating pre- eclampsia and eclampsia according to hospital policy | Y N |  |
| **Patients’ rights are respected** |  |  |
| 1. Rights of women (access to care, information, emotional support, women-centered care, dignity and respect, privacy, companionship) are respected | Y N |  |
| **Interventions of proven effectiveness in place to prevent pre-eclampsia** |  |  |
| 1. Calcium supplementation during pregnancy (at doses of 1.5–2.0g elemental calcium/day) is given in all women, but especially to those at high risk of developing pre-eclampsia | Y N |  |
| 1. Strict bed rest is not recommended for improving pregnancy outcomes in women with hypertension (with or without proteinuria) in pregnancy | Y N |  |
| 1. Diuretics, particularly thiazides, are not used for the prevention of pre-eclampsia and its complications | Y N |  |
| **Preeclampsia is correctly diagnosed** |  |  |
| 1. Pre-eclampsia and sever eclampsia is correctly diagnosed **(*See Annex 14)*** | Y N |  |
| **Management of mild pre-eclampsia is appropriate** |  |  |
| 1. Women with mild pre-eclampsia are followed up as outpatient twice a week | Y N |  |
| 1. For women with mild pre-eclampsia or mild gestational hypertension at term induction of labour is recommended | Y N |  |
| **Management of severe preeclampsia and eclampsia is appropriate** |  |  |
| 1. Following an eclamptic convulsion, birth of the baby is conducted within 12 hours of the onset of the convulsion | Y N |  |
| 1. If diagnosis of severe pre-eclampsia is confirmed, magnesium sulphate is administered (full IV or IM regime) and continued at least for 24 hours after birth or after the last seizure) | Y N |  |
| 1. If systolic blood pressure remains at 160 mmHg or higher and/or if diastolic blood pressure remains at 110 mmHg or higher, antihypertensive drugs are given, as per national protocols, with the goal of maintaining BP below 150/100 but above lover limits of normal ^WHO-S/IMPAC^ | Y N |  |
| 1. Vital signs (pulse, blood pressure, respiration), reflexes and fetal heart rate are monitored regularly (at least hourly) and woman is never left alone | Y N |  |
| 1. A strict fluid balance chart (monitor the amount of fluids administered and urine output) is maintained | Y N |  |
| 1. Calcium gluconate 10% is readily available to counteract the effect of magnesium sulfate | Y N |  |
| 1. Laboratory investigations like CBC,LFTs and RFTs done to assess disease progression and assist in management. |  |  |
| **Decisions regarding timing and mode of birth are in line with current recommendations** |  |  |
| 1. Induction of labour is recommended for women with severe pre- eclampsia at a gestational age when the foetus is not viable or unlikely to achieve viability within one or two weeks *Note: the gestational age threshold of fetal viability should be agreed locally, based on the availability of resources and local survival rates.* | Y N |  |
| 1. In women with severe pre-eclampsia, a viable fetus and before 34 weeks of gestation, a policy of expectant management is recommended, provided that uncontrolled maternal hypertension, increasing maternal organ dysfunction or fetal compromise is absent and can be monitored | Y N |  |
| 1. In women with severe pre-eclampsia, a viable foetus and between 34 and 36 (plus 6 days) weeks of gestation, a policy of expectant management may be used, provided that uncontrolled maternal hypertension, increasing maternal organ dysfunction or fetal compromise is absent and can be monitored | Y N |  |
| 1. In women with severe pre-eclampsia at term, early delivery is recommended | Y N |  |
| 1. Facility is able to offer spinal anesthesia in case cesarean section is indicated |  |  |
| **Women diagnosed with preeclampsia are correctly managed in the postpartum period** |  |  |
| 1. Women are carefully monitored after birth (close monitoring for women with severe pre-eclampsia or eclampsia) | Y N |  |
| 1. In women treated with antihypertensive medicines antenatally, continued antihypertensive treatment postpartum is recommended | Y N |  |
| 1. Women with severe postpartum hypertension are treated with antihypertensive medicines | Y N |  |
| **Discharge and follow up** |  |  |
| 1. Women are given discharge instructions including education on signs and symptoms of severe pre -eclampsia and to seek care immediately if the occur and the dates for postnatal contacts (*day 3; between day 7 and 14; after 6 weeks*). | Y N |  |
| **TOTAL SCORE for management of pre eclampsia and eclampsia (n/26*100)** | **______%** | ***“grade accordingly in the score chart below*** |

*PRE-ECLAMPSIA AND ECLAMPSIA*

| **1 (VERY POOR)**  **If (No services in place for pre-eclampsia)** | **2 (POOR)**  **If (<50%**  **of all standards are scoring Yes)** | **3 (FAIR)**  **If (50-79% of all standards are scoring Yes)** | **4 (GOOD)**  **If (80-99% of all standards are scoring Yes)** | **5 (VERY GOOD)**  **If All standards are scoring Yes)** |
| --- | --- | --- | --- | --- |

| **THE MAIN STRENGTHS AND WEAKNESSES IN THE MANAGEMENT OF PRE-ECLAMPSIA AND ECLAMPSIA** |
| --- |
| **MAIN STRENGTHS:**  **1.**  **2.**  **3.**  **4.** |
| **MAIN WEAKNESSES:**  **1.**  **2.**  **3.**  **4.** |
